# Supplementary material for: Hormonal dynamics and transcriptomic regulatory mechanisms during seed dormancy release in Aconitum kusnezoffii
Source: PLoS One. 2026 Feb 9;21(2):e0340761. doi: 10.1371/journal.pone.0340761 (PMC12885297; doi:10.1371/journal.pone.0340761)
Supplement: Attachment — Submitted filename: PONE-D-25-34985_rev.pdf [file pone.0340761.s003.pdf]

Mu et al. submitted the manuscript dealing with hormonal dynamics and transcriptomic regulatory mechanisms during seed dormancy release in herbaceous perennial plant *Aconitum kusnezoffii*.

Here are my comments:

1. For an unknown reason, the manuscript is written in the MDPI (Agronomy) template. The pages and lines are not numbered, so I refer to the chapter/paragraph titles in my review.
2. **Abstract** – a) in the study, determination of GA_3, ABA, IAA and cytokinin representative zeatin riboside (ZR) was performed. This compound occurs in plant tissue in two isomeric forms, i.e., *trans*- (*t*ZR) and *cis*-ZR (*c*ZR), which differ in their biological activity. It is not specified which isomer of ZR the authors determined. Was it *t*ZR or *c*ZR?
   b) The cytokinins (CKs) generally promote dormancy release. However, the free bases (*t*Z, *c*Z, ...) have greater activity in this direction than corresponding ribosides (*t*ZR, *c*ZR). They are more of a transport form that retains some biological activity, but are not the main actor, although they are able to reversibly release the free base under certain conditions by cleavage of ribose. Thus, it would therefore be more relevant in this case to determine the level of free CK bases rather than CK ribosides.
3. GA_3 is of fungal origin (*Fusarium fujikuroi*) and occurs only to a limited extent in higher plants (http://www.phytohormones.info/ga3refsL.htm). According to this literature source and other sources, GA_3 has not been identified in *Aconitum kusnezoffii* yet. According to the level presented in Figure 2 it could be a contamination from external source. Among 136 known gibberellins (GAs), there are six of them biologically active (GA_1, GA_3, GA_4, GA_5, GA_6 and GA_7). The most common are GA_1 and GA_4 in higher plants. I would recommend to determine the level of other biologically active GA than GA_3.
4. Brassinolide, a representative of bioactive brassinosteroids (BRs), was also performed. Why is not mentioned in an abstract? Further, common abbreviation for this plant hormone is BL, not BR.
5. A) The paragraph "Hormone Quantification lacks the information about sample preparation. How were the hormones extracted from the seeds? How was the crude extract further treated until the sample was pipetted into the ELISA plate?
   B) In the paragraph the authors write that the absorbance was measured. with what instrument? Each of the instruments used should be specified by model name, manufacturer and country of origin. The same applies to chemicals, the list of which should be given in a separate paragraph "Chemicals and Reagents".
   C) Since we do not know the validation parameters of the ELISA kits used, I recommend quantifying plant hormones for a scientific article using some other validated and published method, e.g., LC-MS.
6. Figure 1 is missing a caption.
7. **Results** – a) The paragraph "Dynamic changes of six endogenous hormones during dormancy release…" – Why is there no description of developmental stages in the Material and methods section? Furthermore, why are the developmental stages numbered I, II and IV and not I, II and III?
   b) When expressing the amount of a hormone, it is not necessary to write a dot between the unit (ng/g) and the FW.

Although the manuscript contains partially relevant experimental data, I cannot recommend it for publication in its current form for the reasons mentioned above.
