# Supplementary material for: Hormonal dynamics and transcriptomic regulatory mechanisms during seed dormancy release in Aconitum kusnezoffii
Source: PLoS One. 2026 Feb 9;21(2):e0340761. doi: 10.1371/journal.pone.0340761 (PMC12885297; doi:10.1371/journal.pone.0340761)
Supplement: Attachment — Submitted filename: Response to Reviewers.docx [file pone.0340761.s005.docx]

**Response to Reviewer #1**

*Comment 1: The manuscript is written in the MDPI (Agronomy) template. The pages and lines are not numbered.*
**Response:** We appreciate the reviewer’s comment. The revised manuscript has been reformatted according to the PLOS ONE template, and continuous line numbering has been added for ease of reference.

*Comment 2a: Abstract – ZR is not specified whether it is tZR or cZR.*
**Response:** Thank you for pointing this out. We have clarified in the Abstract and Methods that ZR was quantified by ELISA kits, which cross-react with both trans- and cis-isomers; therefore, the results represent total ZR equivalents.

*Comment 2b: It would be more relevant to determine free CK bases rather than ribosides.*
**Response:** We agree. Due to experimental platform constraints, we quantified ZR as a proxy of CKs. We have added a statement in the Discussion under “Limitations” to acknowledge this methodological limitation and to emphasize that future studies will employ LC–MS to measure free-base CKs (tZ/cZ).

*Comment 3: GA₃ is of fungal origin; GA₃-equivalents might reflect contamination. GA₁ and GA₄ are common bioactive GAs.*
**Response:** We thank the reviewer for this important clarification. In the revision, we have carefully rephrased our interpretation: GA values are reported as GA₃-equivalents (by ELISA) and should be considered proxies of GA-like bioactivity. We explicitly noted in the Discussion that GA₃ has not been confirmed in *A. kusnezoffii*, and future studies will quantify GA₁/GA₄ via LC–MS.

*Comment 4: Brassinolide is not mentioned in the Abstract, and abbreviation should be BL, not BR.*
**Response:** We have revised the Abstract to include brassinolide (BL) and corrected all abbreviations accordingly (BL for brassinolide, BRs for the hormone class).

*Comment 5a: Hormone Quantification lacks details on sample preparation.*
**Response:** We have substantially revised the “Hormone Quantification” section to provide detailed extraction procedures (methanol extraction with PVPP and BHT, centrifugation, N₂ evaporation, PBS reconstitution).

*Comment 5b: Instrument models, manufacturers, and reagents should be specified.*
**Response:** We have added instrument details (e.g., Multiskan FC microplate spectrophotometer, Thermo Fisher Scientific, USA) and created a separate “Chemicals and Reagents” subsection with all suppliers.

*Comment 5c: ELISA kits may not be fully validated; LC–MS is recommended.*
**Response:** We acknowledge this limitation and have clarified that ELISA values are proxies. As suggested, we included a statement in the Discussion noting that future studies will use LC–MS to validate and refine hormone quantification.

*Comment 6: Figure 1 is missing a caption.*
**Response:** We have added the full caption for Figure 1.

*Comment 7a: Developmental stages not defined in Methods; Stages labeled I, II, IV instead of I, II, III.*
**Response:** We have corrected all instances of “Stage IV” to “Stage III” and added a clear “Stage Definition” subsection in the Methods.

*Comment 7b: Units (ng/g·FW) should not contain a dot.*
**Response:** All units have been standardized to “ng g⁻¹ FW.”

**Response to Reviewer #2**

*Comment 1: More details should be given on the number of biological replicates.*
**Response:** We have clarified in “Plant Materials and Dormancy-Breaking Treatment” that each treatment consisted of three independent biological replicates, each with 30 seeds randomly selected from the seed lot.

*Comment 2: The choice of ELISA should be justified.*
**Response:** We have expanded the “Hormone Quantification” section to explain that ELISA was chosen because of its suitability for high-throughput analysis of small seed samples. We also acknowledged its limitations (inability to distinguish isomers, lower accuracy than LC–MS) and committed to applying LC–MS in future work.

*Comment 3: The discussion of ecological and agronomic implications should be expanded.*
**Response:** We have added a new subsection in the Discussion entitled “Ecological and Agronomic Implications,” which highlights the significance of our findings for artificial germination, nursery establishment, and conservation of *A. kusnezoffii*.

*Comment 4: The figures are informative but could be simplified for clarity.*
**Response:** We have simplified selected figures in the revised manuscript to improve readability. The essential hormone dynamics are retained in the main text, and additional data are provided in Supplementary Figures S1–S3.
