# Supplementary material for: Hormonal dynamics and transcriptomic regulatory mechanisms during seed dormancy release in Aconitum kusnezoffii
Source: PLoS One. 2026 Feb 9;21(2):e0340761. doi: 10.1371/journal.pone.0340761 (PMC12885297; doi:10.1371/journal.pone.0340761)
Supplement: Attachment — Submitted filename: Response_to_Reviewers_auresp_2.docx [file pone.0340761.s006.docx]

Response to Reviewer #1

Comment 1:

In the Data availability section, the authors state that the manuscript data have been uploaded to a public database; however, no link or DOI is provided.

Response:
Thank you for pointing this out. We have revised the Data Availability section to explicitly provide the database information and access link. The raw RNA-seq data have been deposited in the NCBI Sequence Read Archive (SRA) under BioProject ID PRJNA1312232, and the direct URL (http://www.ncbi.nlm.nih.gov/bioproject/PRJNA1312232) has now been included in the manuscript.

Comment 2:

The abbreviation BR should be corrected to BL in the Figure 2 caption. In addition, the caption should indicate that ZR values represent the sum of trans- and cis-isomers, and the cross-reactivity of the ELISA antibody should be stated in the text.

Response:
We appreciate this helpful comment. The Figure 2 caption has been revised by replacing BR with brassinolide (BL). The caption now clearly states that ZR values represent the combined sum of trans- and cis-zeatin riboside (tZR + cZR). Furthermore, we have added a clarification in the Materials and Methods section indicating that the ELISA antibody used for ZR quantification exhibits cross-reactivity with both tZR and cZR, and that ZR values are therefore reported as combined equivalents.

Comment 3:

The authors were recommended to add a paragraph in the Materials and Methods section describing the detailed procedure for sample preparation prior to ELISA analysis. This has not been fulfilled. The composition of the extraction reagent, homogenization conditions, centrifugation parameters, and purification using PVPP should be clearly described. Chemical abbreviations should also be explained.

Response:
We thank the reviewer for this important suggestion. We have substantially revised the Materials and Methods section by adding a detailed paragraph describing the complete ELISA sample preparation procedure. This includes the composition of the extraction solvent, homogenization and grinding methods (liquid nitrogen grinding, temperature, and equipment), centrifugation conditions, and purification using polyvinylpolypyrrolidone (PVPP). In addition, all chemical reagents are now fully defined at first mention with their full names followed by abbreviations in parentheses, and a revised *Chemicals and Reagents* subsection has been added to improve clarity and reproducibility.
